# TACE combined with DynaCT-guided MWA in the treatment of high-risk location liver cancers with ≤5 cm tumor size

**DOI:** 10.3389/fonc.2026.1776305

**Published:** 2026-05-07

**Authors:** Daqian Han, Gezhen Wang, Fangzheng Li, Chao Liang, Jiacheng Wang, Yangyang Niu, Hao Li, Shuguang Ju, Manzhou Wang, Xuhua Duan

**Affiliations:** Department of Interventional Radiology, The First Affiliated Hospital of Zhengzhou University, Zhengzhou, China

**Keywords:** high-risk location, liver cancer, microwave ablation, transcatheter arterial chemoembolization, treatment

## Abstract

**Purpose:**

We aimed to investigate the safety and therapeutic effectiveness of the combination of transcatheter arterial chemoembolization (TACE) with DynaCT-guided microwave ablation (MWA) for the treatment of liver tumors ≤5 cm located in high-risk areas.

**Methods:**

The clinical materials of 55 newly diagnosed liver cancer patients with ≤5 cm unifocal lesion admitted to our department from September 2019 to January 2021 were collected. The liver function change, adverse reactions, tumor control rates, cumulative survival rates, overall survival (OS), and progression-free survival (PFS) of the group were compared.

**Results:**

All participants successfully underwent combination treatment with TACE and MWA. According to the Common Terminology Criteria for Adverse Events (CTCAE5.0), adverse events (AEs) and the majority of the complications were classified as grade 1 or 2, involving mild symptoms that required either no intervention or only local, noninvasive management. The mean OS in the high-risk location group was 41.0 months (95%CI = 39.6–42.5), and the mean PFS was 31.2 months (95%CI = 30.7–31.7). The Kaplan–Meier survival curves for OS and PFS are shown in **Figures 1A, B**, respectively. The objective response rate (ORR) 1 month after the first combination treatment in the high-risk location group was 96.4%, and the disease control rate (DCR) was 98.2%. The cumulative survival rates at 6, 12, 24, and 36 months in the high-risk location group were 98.1%, 94.5%, 85.4%, and 74.5%, respectively.

**Conclusions:**

TACE combined with sequential DynaCT-guided MWA may be a safe and promising treatment option for liver cancers ≤5 cm in high-risk locations.

**Figure 1:**
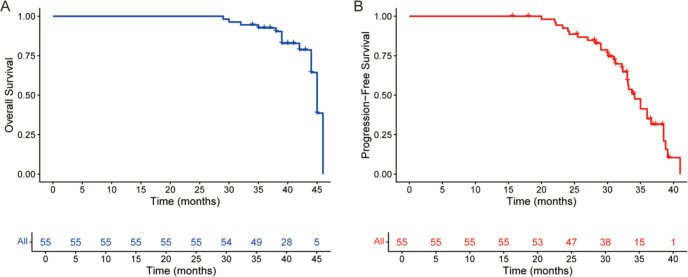
Kaplan–Meier curves of overall survival (OS) **(A)** and progression-free survival (PFS) **(B)** in patients treated with transcatheter arterial chemoembolization (TACE) combined with DynaCT-guided microwave ablation (MWA).

## Introduction

In 2022, an estimated 865,269 new cases of primary liver cancer were reported worldwide, making it the sixth most prevalent malignancy and representing roughly 4.3% of all cancer diagnoses. That same year, it was the third leading cause of cancer-related deaths, responsible for approximately 757,948 fatalities, or 7.8% of the global cancer mortality burden (1). Hepatocellular carcinoma (HCC) comprises approximately 70%–90% of primary liver cancer cases (1). The treatment of HCC involves a multidisciplinary approach, encompassing surgery, systemic therapy, targeted therapy, radiotherapy, and interventional procedures (2). While surgical resection is still considered the primary treatment option for small HCC, a significant number of patients are categorized as high risk due to the presence of concurrent underlying health conditions.

Local ablation therapies, including radiofrequency ablation (RFA) and microwave ablation (MWA), offer clear benefits such as minimal liver injury, reduced invasiveness, and proven effectiveness, providing a potentially curative option for patients who are not candidates for surgical resection (3). The 5-year survival rate after MWA is close to that of surgical resection and liver transplantation, and the efficacy of MWA in the treatment of ≤5 cm liver cancer is equivalent to that of RFA (4). Similarly to RFA, the postoperative prognosis and risk of complications are closely associated with the tumor location (5). A high-risk location tumor is defined as a tumor that is located near the diaphragm dome, intestine, gallbladder, stomach, right kidney, pancreas, large blood vessels, and the subcapsular and other high-risk regions, with the shortest distance between the tumor and the aforementioned organs or lumens being smaller than 0.5 cm (6). The efficacy of MWA alone for tumors in these locations is inferior to that for tumors in non-high-risk locations. Complete ablation is difficult and recurrence rates are high, and severe complications are also more common (7).

Transcatheter arterial chemoembolization (TACE) serves as the standard first-line therapy for patients with advanced, unresectable HCC (8). The combination of TACE and local ablation therapy is frequently employed to enhance treatment efficacy, particularly in patients with tumors located in high-risk areas (9, 10). In a prospective, multicenter study, Peng et al. (11) found that TACE combined with RFA was better than RFA alone in improving the survival rates of liver cancer patients with less than 7 cm lesion. Li et al. (12) used TACE combined with DynaCT-guided MWA to treat small liver cancer (≤3cm) and obtained a significant better efficacy than TACE alone.

In this study, we utilized a combination of TACE and DynaCT-guided MWA (TACE+MWA) to treat patients who had high-risk location liver cancers with ≤5 cm tumors and who were unresectable or unwilling to receive surgical treatment to assess the safety and effectiveness of this combined therapeutic strategy for the treatment of HCC in high-risk locations. Furthermore, this study aimed to evaluate this combined approach in a real-world clinical setting, particularly for patients who are not suitable for surgical resection or for whom ablation alone is technically challenging due to the tumor location.

## Materials and methods

### Clinical data

The patients diagnosed with HCC in this study fulfilled the diagnostic criteria outlined by either the American Association for the Study of Liver Diseases (AASLD) or the European Association for the Study of the Liver (EASL) (13). This study was carried out in compliance with the Declaration of Helsinki and the principles of Good Clinical Practice. The study was reviewed and approved by the Ethics Committee of the First Affiliated Hospital of Zhengzhou University (ethics no. 2023-KY-0736-002). All participants provided written informed consent. This retrospective study included 55 liver cancer patients with ≤5 cm unifocal lesion and who received the combination treatment of TACE plus dynamically DynaCT-guided MWA in our department from September 2019 to January 2021.

The inclusion requirements were as follows: i) Child–Pugh grade A or B; ii) Barcelona Clinic Liver Cancer (BCLC) stage A, or select BCLC stage B patients who were deemed unsuitable for surgical resection or liver transplantation or who declined surgical treatment, particularly in cases where the tumor location made curative ablation technically challenging; iii) Eastern Cooperative Oncology Group performance status (ECOG PS) score of 0–1 within 1 week before enrollment; iv) tumor diameter of ≤5 cm and single; v) expected survival time of >3 months; vi) no evidence of portal vein thrombosis; and vii) no extrahepatic metastases. The following criteria were used to determine exclusion: i) Child–Pugh grade C; ii) BCLC grade C; iii) ECOG PS score >2 within 1 week before enrollment; iv) tumor diameter >5 cm; v) expected survival time <3 months; vi) with portal vein thrombus; and vii) with extrahepatic metastases.

The high-risk location group included 55 patients, among whom 37 were men and 18 were women, with a mean age of 58.63 ± 7.05 years. Among the patients, 42 were infected with hepatitis B, seven were diagnosed with hepatitis C, two patients had Buga syndrome, two patients had alcoholic liver diseases, and the other two patients had other types of hepatitis. Of the patients, 39 presented with tumors ≤3 cm, while 16 had tumors >3 cm in diameter. High-risk location cases included 20 cases close to the diaphragm, 10 cases close to the gallbladder, six subcapsular cases, eight cases in the hepatic caudate lobe, six cases in the hepatic portal region, four cases close to the intestine, and one case close to right kidney. The patient-related variables are summarized in **Table 1**.

**Table 1:** Patient-related variables.

Characteristics	Patients (*n* = 55)	Percentage
Age, mean (range)^a^	58.63 ± 7.05 (42–89)	
≤60	34	61.82
>60	21	38.18
Sex		
Male	37	67.27
Female	18	32.73
Etiology		
Hepatitis B	42	76.36
Hepatitis C	7	12.73
Buga syndrome	2	3.63
Alcoholic liver diseases	2	3.63
Others	2	3.63
Child–Pugh class		
A	51	92.73
B	4	7.27
Maximum diameter (cm)		
≤3 cm	39	70.91
>3 cm	16	29.09
Cirrhosis		
No	20	36.36
Yes	35	63.64
AFP (ng/ml)		
>200	53	96.36
≤200	2	3.64

Data are numbers of patients.

*AFP*, alpha-fetoprotein.

### Procedure

#### TACE treatment

**Figure 2:**
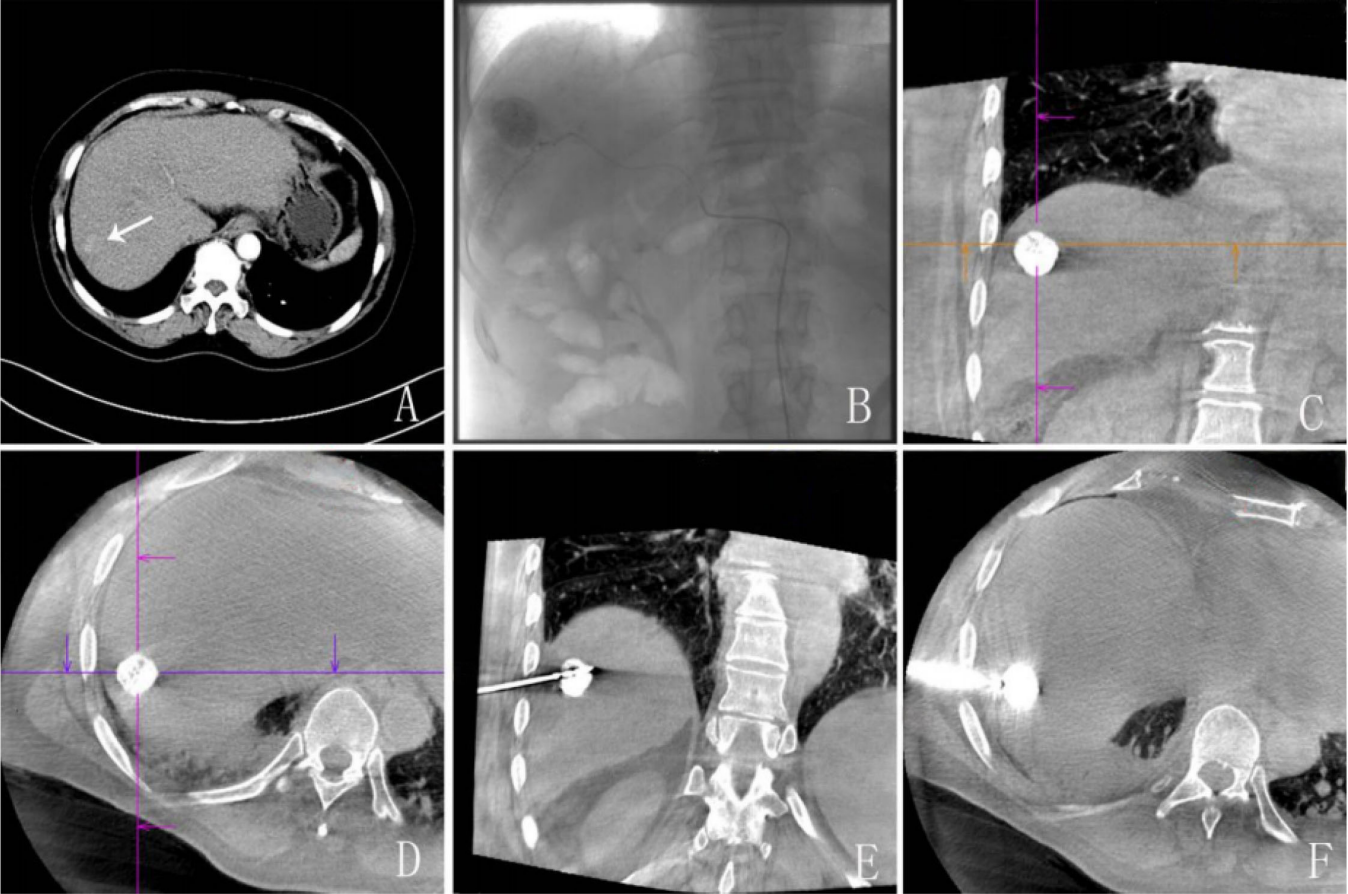
A 61-year-old woman with a 20-year history of hepatitis B and cirrhosis. **(A)** CT showed a 3.3-cm diameter lesion near the diaphragmatic dome, and biopsy showed hepatocellular carcinoma. **(B)** Iodized oil emulsion was used to embolize the blood supply artery of the tumor. **(C)** CT examination after transcatheter arterial chemoembolization (TACE) showed good Lipiodol deposition. **(D–F)** Microwave ablation (MWA) was performed under the guidance of DynaCT 1 week after TACE. Power was set to 60 W and ablated two target lesions for a total of 12 min.

Truncus coeliacus and superior mesenteric artery arteriography was performed via the femoral artery under the guidance of digital subtraction angiography (DSA) (Artis zee BA Twin; Siemens AG, Munich, Germany). When necessary, angiography of the left gastric artery, bilateral diaphragmatic arteries, and intrathoracic arteries was also performed. A mixture of 10–20 ml of iodized oil emulsion and 20–40 mg of doxorubicin or epirubicin was thoroughly emulsified and injected into the tumor-feeding artery until arterial flow stagnation was achieved. Embolization was subsequently reinforced with gelatin sponge particles until complete embolization of the feeding artery was accomplished. After TACE was finished, the patients were scanned with DynaCT (Syngo X-Workplace with Syngo DynaCT; Siemens AG, Munich, Germany) to determine the extent of iodine oil uptake in the tumor (**Figure 2**).

#### MWA treatment

At 5–7 days after TACE, MWA was performed using the same DSA machine. After intravenous or general anesthesia, patient positioning was refined using real-time tumor localization and confirmed through DynaCT imaging. The CT images were collected and automatically uploaded to the Syngo Workplace workstation to reconstruct 3D volumetric images and axial, sagittal, and coronal multiplanar reconstruction (MPR) images to assist puncture planning. Under the guidance of DynaCT, according to the iGuide 3D puncture localization technology, the liver cancer lesions that had been embolized were punctured. Specific procedures referred to our previous study [9]. During the puncture, color Doppler ultrasound (LUS; ALOKA ProSound α5, Aloka, Tokyo, Japan) was used to observe whether the MWA electrode needle (ECO-100AI10; ECO Microwave System Co., Nanjing, China) passed or was adjacent to larger bile ducts or blood vessels.

**Figure 3:**
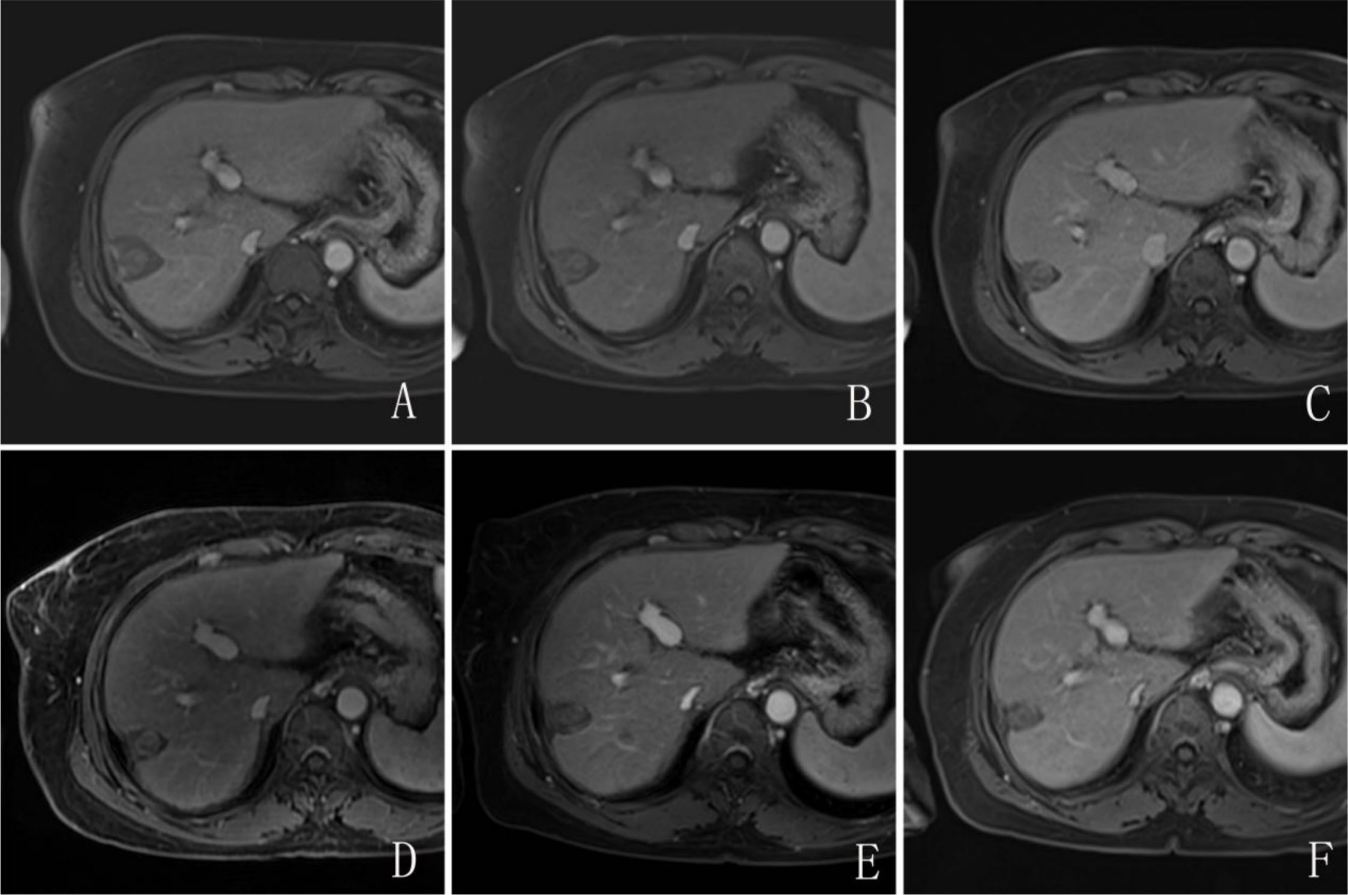
The same patient as in **Figure 2**. MRI re-examination at 1 month **(A)**, 3 months **(B)**, 6 months **(C)**, 1 year **(D)**, 2 years **(E)**, and 3 years **(F)** after the combined transcatheter arterial chemoembolization and DynaCT-guided microwave ablation (TACE+MWA) procedure did not show obvious enhanced lesions.

The tumor ablation strategy was as follows: deep lesions were ablated first, followed by superficial lesions. For lesions near the gallbladder, intestine, or other hollow viscera, artificial ascites or pleural effusion was selectively considered, but was rarely used due to anatomical limitations, adhesions, or patient tolerance. Safety was ensured through careful pre-procedural planning, precise puncture path selection along the long axis of adjacent organs, and operator experience. Output power and the ablation time were adjusted according to the tumor size (generally 40–70 W), while the ablation range was planned to extend approximately 0.5 cm beyond the tumor edge. Due to the lack of intra-procedural contrast in DynaCT, the ablation margin was verified using a combination of pre-procedural imaging, Lipiodol deposition from prior TACE as a tumor marker, and the 3D reconstructed images from the Syngo Workplace. When withdrawn, the needle was heated to ablate the needle canal in order to prevent bleeding and tumor spread. Based on the tumor size, one-time *in situ* ablation was used for ≤3 cm tumors, while two to four ablations were used for tumors of 3–5 cm. After ablation was completed, the puncture site was pressed for 10 min. If necessary, ultrasound was used to check for bleeding and biliary fistula (**Figure 3**).

### Efficacy evaluation

The primary outcome measures of this study were overall survival (OS) and progression-free survival (PFS). The modified Response Evaluation Criteria in Solid Tumors (mRECIST) was used to evaluate the treatment effect. Complete remission (CR): re-evaluation of the enhanced CT and/or MRI plain scan + dynamic enhancement showed that the enhancement of the target lesion in the arterial phase disappeared; partial remission (PR): the total diameter of the enhanced target lesions in the arterial phase decreased by ≥30%; stable disease (SD): the shrinkage of lesions did not reach PR or increased, but below the level of progression; progression disease (PD): the total diameter of the enhanced target lesions in the arterial phase increased by ≥20% or new lesions appeared. The objective response rate (ORR) was calculated as: ORR = (number of CR cases + number of PR cases)/total × 100%. The disease control rate (DCR) was calculated as: DCR = (number of CR cases + number of PR cases + SD cases)/total × 100%. PFS was defined as the time from the start of treatment to the progression of the disease or death from any cause. OS was defined as the time from the start of treatment to death for any reason or the follow-up deadline. To accurately distinguish residual viable tumor from Lipiodol deposition, all post-treatment enhanced CT and/or MRI images were independently reviewed by two experienced interventional radiologists. Residual tumor was defined as arterial phase enhancement with washout in the portal or delayed phase, whereas dense Lipiodol deposition without enhancement was considered non-viable. Any discrepancies were resolved by consensus. This approach ensured objective and accurate assessment of treatment response, including the ORR, CR, and DCR.

### Follow-up indicators and further treatment

Laboratory tests such as alpha-fetoprotein (AFP) and liver function tests were performed before and after operation. Adverse reactions such as nausea, vomiting, pain, pleural fluid or ascites, gastrointestinal bleeding, and perforation during and after the operation were recorded. Postoperative complications were promptly managed. Abdominal enhanced CT and/or MRI was re-evaluated 1 month after the operation to record the extent, range, and local recurrence of tumors. For patients undergoing initial treatment with TACE combined with MWA, radiographic evaluation should be conducted according to the mRECIST: those achieving CR will enter systematic follow-up monitoring, whereas cases evaluated as PR should receive continued combination therapy with the cumulative number of treatment sessions strictly limited to three, followed by transition to periodic follow-up observation.

### Statistical methods

SPSS 21.0 statistical software was used for statistical analysis. Quantitative data were expressed as the mean ± standard deviation (mean ± SD). Qualitative data were expressed as percentage. The Kaplan–Meier method was used to plot the OS and PFS survival curves. Log-rank test was used to compare the survival curves. Univariate Cox proportional hazards regression models were used to analyze the prognostic factors for PFS and OS. Due to the relatively small sample size (*n* = 55) and the low number of events, multivariate Cox regression including multiple covariates was not performed as this could result in overfitting and unreliable estimates. A *p* < 0.05 was considered to be statistically significant.

## Results

The median follow-up duration was 36.0 months. All patients completed one to three courses of the combination treatment after 6 months, until CR, PR, or intolerance to the treatment. Patients in the high-risk location group were treated for 1.34 ± 0.55 times. Although patients experienced different degrees of postoperative liver damage, liver function could recover to essentially normal levels within approximately 2 weeks. Liver function was assessed according to the Child–Pugh scoring system. There was no significant difference between the preoperative Child–Pugh score and the Child–Pugh score 6 months after the first treatment (all *p* > 0.05). Postoperative complications included fever, nausea, vomiting, and pain, the majority of which were mild and self-limiting. No major complications requiring invasive interventions (e.g., drainage, surgery, or intensive care) were observed. Abdominal and pleural effusions were small in volume and resolved with conservative management. There were no major bleeding and death cases associated with TACE or MWA (**Table 2**).

**Table 2:** Adverse events and complications (*n*, %).

Category	Patients (*n* = 55)	Percentage
Adverse events		
Fever	46	83.60
Nausea or vomiting	33	60.00
Mild pain, requiring non-opioid oral analgesic treatment	25	45.50
Moderate pain, requiring opioid oral analgesic treatment	4	7.30
Mild liver dysfunction, requiring conservative treatment	2	3.60
Total bilirubin elevation, transient	2	3.60
Hypoalbuminemia, transient	2	3.60
Complications		
Abdominal effusion	5	9.10
Pleural effusion	2	3.60

All adverse events and complications were classified as mild (grades 1–2) according to the Common Terminology Criteria for Adverse Events (CTCAE v5.0) and were managed conservatively without the need for invasive intervention. The abdominal and pleural effusions were small and self-limiting, requiring no drainage.

### Treatment efficacy

The patients were evaluated by imaging examination 1 month after the first treatment. In the high-risk location group, the cases of CR, PR, SD, and PD were 38, 15, 1, and 1, respectively. The ORR and DCR were 96.4% and 98.2%, respectively. Re-evaluation 6 months after the first treatment showed that, in the high-risk location group, the cases of CR, PR, SD, and PD were 43, 8, 4, and 0, respectively. The ORR was 92.7%, while the DCR was 100%.

In the high-risk location group, the cumulative recurrence rates after 12, 24, and 36 months were 12.7%, 29.1%, and 47.3%, respectively. The 6-, 12-, 24-, and 36-month cumulative survival rates were 98.1% (54/55), 94.5% (52/55), 85.4% (47/55), and 74.5% (41/55), respectively. There were eight patients who died from tumor recurrence and metastasis, five patients who died from liver failure, and one patient who died from gastrointestinal major bleeding. In the same group, the average OS was 41.0 months (95%CI = 39.6–42.5 months), while the average PFS was 31.2 months (95%CI = 30.7–31.7 months) (**Figure 1**). Univariate Cox proportional hazards regression indicated that sex (male *vs*. female), age (>60 *vs*. <60), Child–Pugh class (A *vs*. B), and etiology (hepatitis B or C *vs*. others) were not associated with PFS and OS (all *p* > 0.05) (**Table 3**).

**Table 3:** Factors affecting progression-free survival (PFS) and overall survival (OS).

Parameter	OS	PFS		
	HR (95%CI)	*P*-value	HR (95%CI)	*P*-value
Univariate Cox regression				
Sex (male *vs*. female)	2.95 (0.90–9.79)	0.077	1.69 (0.59–4.88)	0.333
Age (≥60 *vs*. <60)	0.71 (0.23–2.19)	0.549	1.20 (0.41–3.50)	0.733
Child–Pugh class (A *vs*. B)	0.33 (0.04–2.65)	0.294	1.81 (0.40–8.13)	0.437
Etiology (yes *vs*. no)	2.63 (0.55–12.70)	0.228	1.44 (0.32–6.46)	0.636
AFP (ng/ml) (≥200 *vs*. <200)	3.45 (0.43–27.75)	0.244	2.67 (0.34–20.77)	0.348
Maximum diameter (cm) (≤3 cm *vs*. >3 cm)	2.14 (0.58–7.98)	0.255	6.40 (2.00–20.45)	0.002
Multivariate Cox regression				
Sex (male *vs*. female)	–	0.072	–	0.208
Age (≥60 *vs*. <60)	–	0.505	–	0.206
Child–Pugh class (A *vs*. B)	–	0.268	–	0.865
Etiology (yes *vs*. no)	–	0.222	–	0.558
AFP (ng/ml) (≥200 *vs*. <200)	–	0.215	–	0.386
Maximum diameter (cm) (≤3 cm *vs*. >3 cm)	–	0.268	6.28 (1.96–20.05)	0.002

En dash denotes that multivariate Cox regression was only performed for tumor maximum diameter. Other variables were not included due to the limited sample size and low event rates.

*HR*, hazard ratio; *AFP*, alpha-fetoprotein.

## Discussion

MWA has advantages of high ablation efficiency, short ablation time, and a reduced “heat sink effect.” In addition, MWA is not affected by metal substances in the body, providing ablation opportunities for patients undergoing stent or pacemaker implantation (14). A prospective pilot study showed no significant differences between MWA and RFA in terms of local efficacy, complication incidence, and survival rates (4). A randomized controlled trial (RCT) compared RFA and MWA in the treatment of ≤5 cm liver cancer, which found that MWA could reduce the number of punctures and ablation time, has a wider indication range than RFA, and is more effective for 3- to 5-cm tumors in high-risk locations, e.g., adjacent to the gallbladder or large blood vessels (15). However, during stand-alone MWA procedures, the assistance of artificial surgical ascites is typically required to facilitate surgical manipulation. This technique presents two major limitations: prolonged procedural duration on the one hand and a relatively poor tolerance to the thermal ablation stimuli in the diaphragmatic dome tissue on the other hand, predisposing patients to postoperative complications.

In terms of treatment strategy, according to the current BCLC guidelines (16), surgical resection and local ablation are recommended as the first-line curative options for early-stage HCC (BCLC stage A), while TACE is generally recommended for intermediate-stage disease (BCLC stage B). However, in real-world clinical practice, treatment decisions are often influenced by the tumor location, the technical feasibility, and the patient conditions. In particular, for tumors located in high-risk anatomical regions, ablation alone may be associated with an increased risk of incomplete treatment and complications, and surgical resection may not always be feasible. Therefore, select patients with BCLC stage A or B disease may benefit from a combined approach such as TACE+MWA. Our study reflects this treatment strategy in a real-world setting and supports its potential role as an alternative option in carefully selected patients.

Liver cancers in high-risk locations are difficult to treat using both surgical resection and ablation due to their unique locations. The incidence of postoperative complications and the rates of recurrence are higher, and middle- or long-term survival is poor (17). Liver cancers in high-risk locations have a relatively high incidence, accounting for 23.4%–34.0% of liver cancer cases (18, 19). Therefore, it is of great value to determine a suitable treatment plan to improve the efficacy and avoid serious complications. Several strategies have been applied to achieve this goal. Artificial pleural effusion or artificial ascites technique is used to assist ablation for subdiaphragmatic lesions or lesions adjacent to the gallbladder or the digestive tract (20, 21). For subcapsular lesions, the ablation needle passes through a section of liver tissue to enter the target lesion. After ablation, the needle is retreated slowly and the needle canal is ablated when retreating the needle, which can reduce the incidence of bleeding and implant metastasis (20). For lesions in the hepatic portal region, blocking the blood flow can reduce the heat sink effect of large blood vessels (22). Ablation can be combined with TACE (8) or percutaneous ethanol injection (PEI) (23) to improve efficacy. Sequential treatment with TACE combined with 3D visualization ablation planning system-assisted RFA can be used for lesions in high-risk locations. In this study, we used the combination of TACE and dynamically DynaCT-guided MWA, which is the same as that used by Li et al. (12).

Song et al. (24) used ultrasound-guided RFA to treat ≤5 cm liver cancer near the diaphragm and intestine. The 1- and 2-year survival rates were 97.2% and 97.2%, respectively, and the median tumor-free survival was 16.1 months. Kelogrigoris et al. (25) used CT-guided RFA to treat high-risk location liver cancers with diameters of 1.5–6 cm. The 1-, 2-, and 3-year survival rates were 82.6%, 67.3%, and 54.1%, respectively. In the study by Wong et al. (26), RFA combined with PEI was used to treat high-risk location liver cancers. The 1-year survival rate was 87%, while the 2-year survival rate was 77%. Yu et al. (27) treated ≤5 cm liver cancers using MWA. The 1-, 3-, and 5-year intrahepatic metastasis rates were 3.5%, 22.9%, and 58.7%, respectively, while the extrahepatic metastasis rates were 1.6%, 5.9%, and 13.2%, respectively. The 1-, 3-, and 5-year survival rates were 96.4%, 81.9%, and 67.3%, respectively. Similar to the above studies, we also applied a combination treatment strategy in this study. With one to three treatment sessions, patients with ≤5 cm unifocal lesions achieved survival outcomes comparable to those reported in previous studies, suggesting that TACE combined with MWA may be an appropriate treatment option. However, such comparisons should be interpreted with caution due to the differences in patient selection, the definitions of high-risk locations, and the treatment eras. Compared with other ablation-based combination strategies, TACE combined with DynaCT-guided MWA may offer advantages in lesion visualization and targeting accuracy, as Lipiodol deposition helps delineate the tumor boundaries and 3D guidance facilitates precise puncture in complex anatomical regions. In addition, TACE may reduce the heat sink effect and enhance the ablation efficacy. However, limitations such as the lower image resolution of DynaCT and the lack of intra-procedural contrast may affect margin assessment, and the technical complexity of the procedure may limit its widespread applicability. In addition, several practical considerations should be taken into account. The implementation of DynaCT requires specialized equipment, which may increase economic costs and limit its availability in some centers. Furthermore, the sequential combination of TACE and MWA involves a two-step procedure, potentially increasing resource utilization and procedural time. The technique also requires a certain level of operator experience, and the associated learning curve may further affect its broader adoption in routine clinical practice.

With regard to the combination of TACE and RFA, although TACE embolizes tumor vessels to ensure that the ablation lesions reach the required temperature, when the lesions are close to large blood vessels, the residual tumor tissue may not be heated enough to necrosis due to the “heat sink effect,” leading to local recurrence (28). As for TACE combined with PEI, when the tumor diameter is greater than 3 cm, PEI requires multiple puncture ablations, increasing the risk of metastasis, with the residual tumor tissue giving rise to local recurrence (29). TACE combined with CT-guided MWA has achieved good efficacy for ≤3 cm small liver cancer. A number of scholars (12, 30) believe that this combination treatment has the following merits: 1) TACE blocks the arterial blood supply of liver cancer; 2) majority of the tumor tissues are damaged and the diameter is reduced, which is conducive to subsequent sequential ablation treatment; 3) the tumor blood supply arteries are occluded and the “heat sink effect” is significantly reduced; 4) the ischemia and hypoxia of tumor cells after TACE increase their heat sensitivity so that a lower temperature can kill them; and 5) MWA can supplement the area where TACE cannot completely embolize and improves the complete clearance rate of tumor. In addition, TACE has a tumor-marking effect, and the Lipiodol injected during TACE can be visualized on CT scans, delineating the original tumor boundaries and facilitating subsequent MWA targeting. In certain patients, the tumors shrink after TACE, and the surrounding fibrous encapsulation enhances boundary recognition. Therefore, the combination of TACE and MWA takes advantages of each other’s strengths and overcomes the shortcomings of other combined therapies.

In this study, TACE and MWA were sequentially applied for the treatment of liver cancer. The main complications were post-embolization syndrome after TACE and pain during MWA ablation, as well as post-ablation syndrome. As the tumor size was small, the main symptoms of post-embolism syndrome including fever, pain, nausea, and vomiting were relatively mild. During the MWA, there was no obvious intraoperative pain owing to appropriate analgesic management. There were mild and severe postoperative complications. A common postoperative complication is post-ablation syndrome, which has symptoms of fever, nausea, pain, and ascites formation. Severe complications include subcapsular hemorrhage, gastrointestinal bleeding, liver abscess, diaphragmatic perforation, peritonitis, and implantation metastasis following liver puncture.

TACE was performed under the guidance of DSA. Images were collected with a DynaCT scan. Through the use of the 3D iGuide puncture technique, the CT images could be reconstructed at the workstation for use in designing the puncture site and path and in guiding the puncture subsequently, so that TACE and ablation can be conducted on the same DSA machine (12). DynaCT clearly delineates the tumor boundaries, necrotic areas, and the spatial relationship with adjacent critical structures. In our study, despite various locations of the tumors, TACE combined with dynamically DynaCT-guided MWA could be accurately and safely conducted, suggesting that this combination treatment could achieve favorable efficacy across different tumor locations.

In this study, almost half of the high-risk location cases were close to the diaphragm. Artificial pleural effusion or ascites was rarely used to assist the ablation of lesions adjacent to the diaphragmatic dome, liver capsule, gallbladder, and cavity organs in our study. Firstly, in a number of patients, due to abdominal adhesions or anatomical limitations, it is impossible to create effective artificial ascites, leading to failure of the procedure, which places high technical demands on the operator. Secondly, the preparation of artificial pleural and abdominal effusion requires additional procedural steps, which can prolong the operation time and increase the risk of anesthesia. Finally, the injection of artificial pleural and abdominal effusion will increase the burden on the heart and lungs, and some patients may have difficulty tolerating this.

Our study has several limitations. DynaCT requires real-time monitoring of the needle puncture, which increases X-ray exposure. In addition, its image resolution is lower than that of conventional CT, making it more challenging to distinguish subtle tissue differences. Moreover, intraoperative contrast enhancement is not possible, which may affect the accuracy of ablation. Although we estimated a 0.5-cm ablation margin using pre-procedural imaging, Lipiodol deposition from TACE as a tumor marker, and 3D reconstruction guidance, some local recurrences may still be related to these inherent limitations. This study is also a single-center, retrospective investigation with strict inclusion criteria, such as solitary tumors ≤5 cm, preserved liver function (Child–Pugh A or B), and absence of extrahepatic metastasis, which may have introduced selection bias. Consequently, our findings primarily reflect a highly select patient population and may not be fully generalizable to a broader, more heterogeneous real-world cohort. The relatively small sample size and the heterogeneity of the high-risk tumor locations further limit interpretation. These locations include the diaphragm, gallbladder, liver capsule, caudate lobe, hilum, intestine, and kidney, each presenting unique technical challenges. Pooling such diverse subgroups may mask differences in outcomes and complications. In particular, data on local tumor control, complication rates, and survival outcomes stratified by specific high-risk anatomical locations (e.g., subdiaphragmatic, perihilar, or adjacent to bowel) were not analyzed. Due to the limited number of patients in each subgroup, such analyses were not statistically feasible, which limits the ability to evaluate the applicability of this approach across different high-risk clinical scenarios. Another consideration is that artificial ascites or pleural effusion was rarely used for tumors adjacent to hollow viscera, such as the gallbladder or intestine, due to anatomical constraints, adhesions, or patient tolerance. The absence of major complications likely reflects the careful patient selection, meticulous procedural planning, and operator experience rather than generalizable safety. In addition, the study did not include a control group receiving TACE alone, MWA alone, or MWA guided by other modalities, limiting direct comparisons of the efficacy and safety of this combination therapy. Therefore, while our results suggest that the combination of TACE with DynaCT-guided MWA may be safe and effective in this high-risk cohort, these findings should be interpreted with caution. Future multicenter, prospective studies with larger and more diverse patient populations, including subgroup analyses, control groups, and evaluation of protective techniques, are needed to rigorously validate the safety and efficacy of this combination therapy in high-risk liver cancers.

## Conclusion

This study suggests that TACE combined with sequential DynaCT-guided MWA may be a safe and promising treatment option for liver cancers ≤5 cm in high-risk locations. However, these findings should be interpreted with caution and require further validation in larger, prospective, controlled studies.

## Data Availability

The original contributions presented in the study are included in the article/supplementary material. Further inquiries can be directed to the corresponding author.
